# Incorporating immersive learning into biomedical engineering laboratories using virtual reality

**DOI:** 10.1186/s13036-022-00300-0

**Published:** 2022-08-08

**Authors:** Megan Wilkerson, Vitali Maldonado, Srikanth Sivaraman, Raj R. Rao, Mostafa Elsaadany

**Affiliations:** Department of Biomedical Engineering, University of Arkansas, Fayetteville, USA

**Keywords:** Virtual reality, Biomedical engineering laboratory, STEM remote learning, Engineering education

## Abstract

**Background:**

The Covid-19 pandemic caused a sudden shift towards remote learning, moving classes to online formats. Not exempt from this switch, laboratory courses traditionally taught in-person were also moved to remote methods, costing students the opportunity to learn these skills hands-on. In order for instructors to provide course materials effectively and engagingly, non-traditional methods should be explored.

Virtual reality (VR) has become more accessible in recent years. VR simulations have been used for many years as educational tools in high-risk settings such as flight or medical simulations. Immersive VR videos implemented in a remote laboratory course could provide the students with an engaging and suitable learning experience.

To test the effectiveness of VR videos as a tool for remote education, VR videos of the laboratory component of a Biomolecular Engineering course were provided to students. A survey was distributed for students to self-report their experience with the videos. The survey contained quantitative and qualitative ratings of VR as an educational tool.

**Results:**

The survey showed that students (~ 89% strongly agree or agree) believed the videos provided the opportunity to work at their own pace and were an appropriate length. While ~ 74% of students said that the videos provided enough information to understand the tasks, a small percentage felt that the videos improved their retention (~ 16%) and understanding (~ 9%) of the course material. About 28% of the students responded positively when asked about how VR videos improved their engagement with the material. ~ 30% reported confidence in applying the skills learned in the videos in the future and ~ 43% believe the VR videos were an acceptable alternative to in-person labs. Two-thirds of students reported feeling some form of discomfort while viewing the VR videos and 54% reported not using the headset for the videos and using the 3D video feature instead.

**Conclusions:**

As many students reported the videos containing appropriate information, the content of the videos was not an issue. A combination of improved camera quality with motion stability, more comfortable headsets, and a reduction in editing issues could greatly improve the quality and effectiveness of VR videos.

## Background

The Covid 19 pandemic caused a massive move to remote instruction throughout the education system from primary to higher education. A wide variety of methods for delivering courses have been adopted with some courses continuing live classes over video conferencing applications and others opting for asynchronous instruction [1]. Online classes have already been established as an alternative to traditional face-to-face courses for students who cannot be on campus. However, these classes were designed to be delivered and completed remotely. Classes typically delivered in person had to alter the structure and requirements of the class in a short amount of time, a difficulty for both teachers and students. These classes have struggled to present course material in ways that the students view positively. Students have found the remote methodologies to be difficult in a study by Bawa (2020) [2]. The study showed that 66% of students had negative comments about the transition to remote learning. The second and third most common topics of comments are the difficulty in learning due to this transition, and difficulty in learning from online materials respectively [2]. In order to provide a learning experience that conveys the material while keeping the students engaged and focused on the lesson, non-traditional methods need to be explored.

Virtual reality (VR) has become more accessible in the last few years, not only in terms of experience but also in terms of design. Various cameras, editing software, and headsets are available to create and view VR environments. VR simulations have already been used for many years for flight simulations and medical simulations, providing a safe way to gain experience in higher-risk situations [3]. VR environments provide a sense of presence [4] as they provide an immersive experience. Additionally, multiple studies showed that the implementation of VR technologies in the scholarly agenda had positive outcomes [5–7]. Often these VR environments use head-mounted displays allowing the user to look around the scene as they would in reality. The use of VR videos shows promising yet mixed results when implemented in educational settings [8]. Several studies have looked into involving VR in biomedical engineering education. In a study by Singh et al. (2020) [9], surveys of students immersed in 3D VR modules and their learning objectives were compared with students subjected to 2D videos. In addition, the student feedback indicated that students preferred 3D VR modules over 2D videos as they enhanced their experiential learning. The surveys also indicated that 3D VR modules were superior to 2D videos in simulating real-world scenarios more closely. However, studies have also suggested that 3D VR modules and 2D videos have similar learning outcomes [10]. Great care must be taken when designing the VR environment to receive the greatest benefits. Immersive VR lessons containing too many additional graphics or distractions not relevant to the course material may cause a negative effect on students’ learning [11]. It is important that the virtual environment must be focused on the material being taught.

Laboratory components are essential to STEM courses as they provide an opportunity for students to learn skills hands-on that they can apply later in their careers [12]. Traditionally, labs are conducted in person where students get to use the equipment and techniques to achieve the targeted outcome. To aid the learning process, an instructor is usually present who can demonstrate and guide the students. This valuable learning experience may be lost when students cannot be present in the lab.

To make up for the inaccessibility of some labs, videos or simulations of lab procedures have been developed and used successfully [13]. Similar videos are also commonly used to supplement traditional teaching methods to provide greater context and visual representations of concepts. Providing supplemental videos for students to refer back to throughout the course allows students to fill gaps in their understanding that were missed initially [14]. Applying this practice to lab demonstrations enables students the opportunity to watch how to perform laboratory tasks additional times as opposed to limited iterations during an in-person lab. Additionally, in-person labs generally have many students per instructor, possibly causing the instruction to be less effective for some students. Virtual labs provide students the ability to be at the center of the instruction that can contribute to greater learning [15].

Remote labs utilizing VR could provide an added impact of greater engagement with the lab materials. Engagement is the investment of effort and attention towards the material. Developing more engaging methods of conveying content creates a positive view and greater perceived understanding of the material in students [16]. In the case of online education, student engagement relies on the students' ability to participate and be present during the lesson [17]. An immersive VR lab would focus the students' attention on the lab task while providing a sense of being present in the lab.

Previous studies have used student self-reported surveys as the method of data collection to gather students’ perceptions of their educational environment. Brown et al. (2016) [18] used student self-reported surveys to gather data on students’ perception of STEM courses and their believed self-efficacy in STEM courses. Sandi-Urena (2020) [19] surveyed students about the remote chemistry lab experience using a survey for students to report what opportunities they had to experience course expectations and their satisfaction with the way that expectation was delivered. Pagano et al. (2018) [20] and Indorf et al. (2019) [21] utilized CURE surveys (course-based undergraduate research experience) in STEM courses to understand students' perceived learning gains, understanding, and persistence in STEM courses. Wilson et al. (2015) [22] used student self-reported surveys to study the connections between STEM students’ sense of belonging at their institution and their engagement in STEM courses. Finally, Choudhury (2019) [23] used student surveys to understand how to improve engineering education.

VR shows great potential as an educational tool. This study aims to assess VR as an educational tool for a remote laboratory component in an undergraduate biomedical engineering course. We hypothesize that VR videos could be an effective substitute for lab courses when laboratory spaces are inaccessible. Student engagement with the material and the ability of VR videos to convey technical knowledge are measured using student feedback from distributed surveys.

## Materials and methods

To measure the efficacy of VR as an educational tool, VR videos were distributed to students in an undergraduate junior-level biomolecular engineering laboratory course. The sample group consisted of 56 participants, roughly between 18 and 25 years old, in their third year or higher of undergraduate studies and enrolled in the (College of Engineering) Biomedical Engineering department. All participants consented to participate in this study. Four lab modules implemented VR videos. The videos contained lab procedures, experiments, and theory that complemented the biomolecular engineering course schedule. Moreover, the videos contained images to imitate the PowerPoint slides that would have been presented in person. These videos lasted between 20–50 min. Students attended a remote lab session to allow the teaching assistants (TAs) to clarify aspects of the lab procedures featured in the videos. Pre- and Post-lab quizzes were utilized for comparison between lab modules with and without the VR videos. Finally, students took a survey regarding VR videos. This study was reviewed by the University’s Institutional Review Board (protocol #: 2012306663) and was determined to be exempt.

### Experimental design

Students were provided with VR videos as an alternative to in-person labs. All students watched the same lab videos to ensure equal quality and access to materials. For each lab, students were provided the lab protocol, the lab video, and data to analyze. Students were able to read the lab protocol before watching the lab video to gain an understanding of the purpose and procedures of the lab. Students were provided the VR lab videos to view prior to their lab sessions. Pre-lab quizzes were administered to students after watching the lab videos, but before they attended the remote lab sessions. Lab sessions with the TAs were held over Zoom during the students’ scheduled lab time for the students to discuss the lab with the TAs and begin writing a lab report. After the lab meeting, students had until their next lab meeting to take a post-lab quiz.

A survey was distributed once at the end of the semester, after the completion of all the lab modules, to all the students taking the lab as part of their course schedule that semester. A mixture of 5-point Likert scale (where 1 = Strongly Disagree, 3 = Neutral and 5 = Strongly Agree) and free-response questions were used. The objective of this survey was to measure the effectiveness of VR videos as an educational tool through student feedback.

### Video creation

**Fig. 1:**
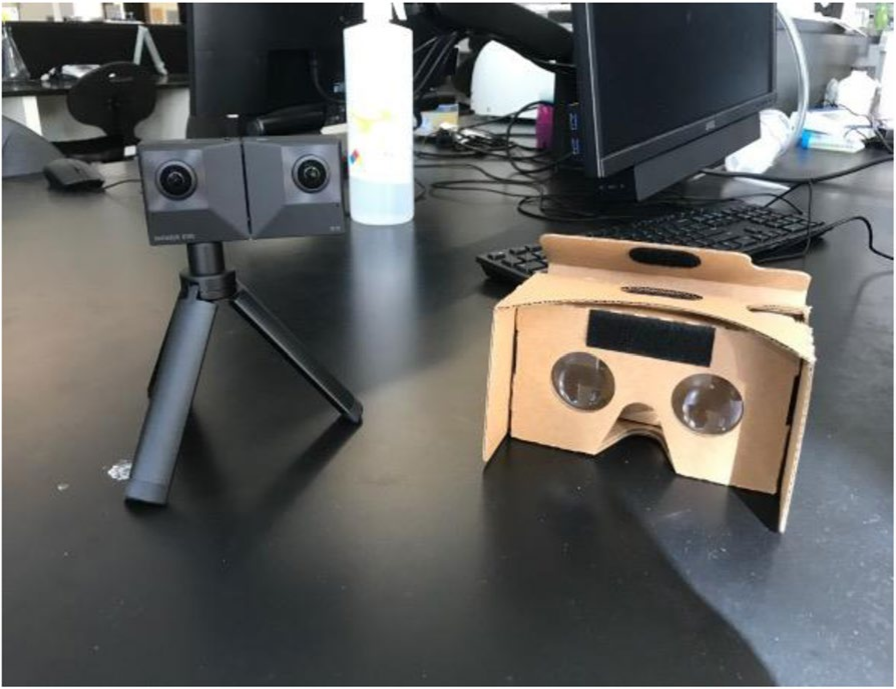
The Insta360 EVO VR camera in 180° 3D mode and the Google Cardboard headset folded into viewer position

**Fig. 2:**
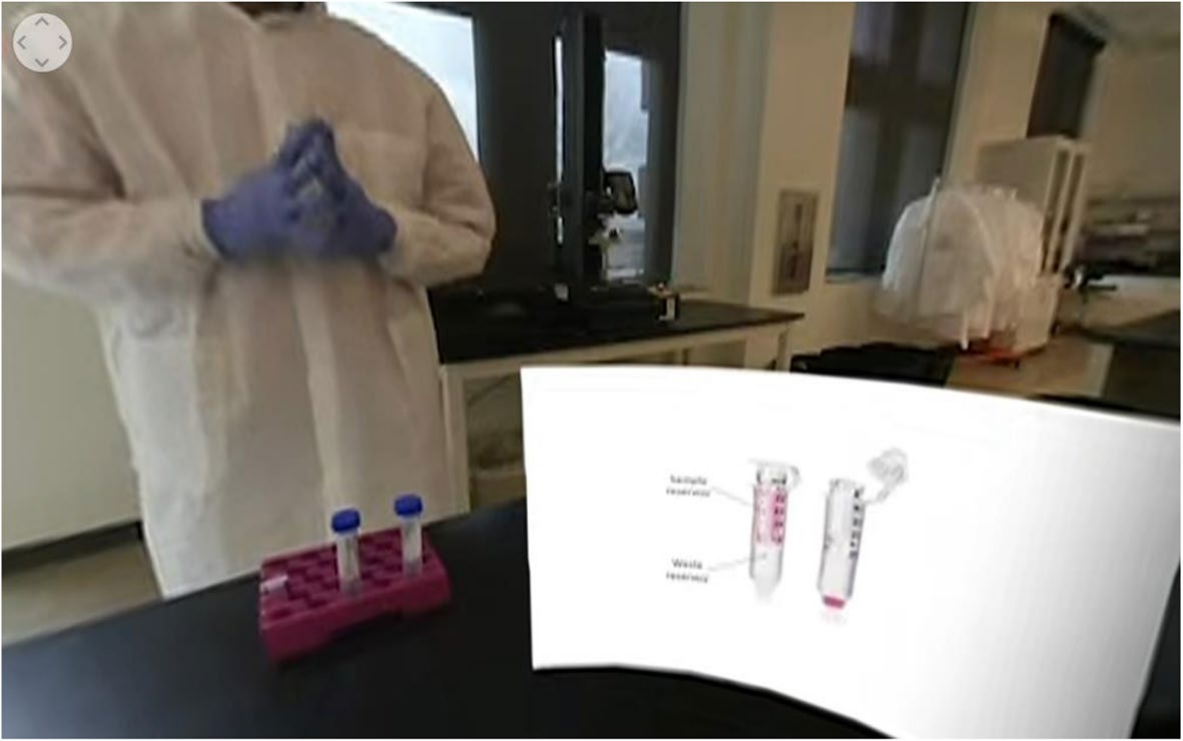
Screenshot of a VR lab video displaying a diagram of a centrifugal filtration unit

Laboratory experiments for an undergraduate biomolecular engineering course laboratory component were filmed to be implemented as a remote lab experience. The videos were filmed using an Insta360 EVO camera (Fig. 1, left). This camera has two 180° lenses that allow for recording in 360° or 180° with a 3D effect. All the lab videos were filmed in 180° 3D to add dimension to the VR video and to remove possible distractions from the unnecessary 180° behind the camera. The videos were edited using Adobe Premiere Pro. Images or text were inserted in some of the videos to represent what the instructor was explaining, such as a diagram of a centrifugal filtration unit with the sample reservoir and waste reservoir components labeled, as seen in Fig. 2.

The videos were uploaded to YouTube and accessed by students through a link provided on the university’s learning management system (Blackboard). YouTube provides a platform for VR videos to be watched using a headset or a desktop computer. On a desktop, the video will appear similar to a regular video, but it has the ability for the user to click and drag the view of the video around the 180° space, providing a partial VR effect if the viewer cannot use a headset. Students were given Google Cardboard headsets to view the VR videos (Fig. 1, right). The Google Cardboards are made of cardboard with a set of lenses to create the VR effect. For the display screen, the students navigate to the YouTube video on their smartphone, select the VR button to enter the phone into a VR viewer mode, and insert their phone into the cardboard viewer.

### Survey

**Fig. 3:**
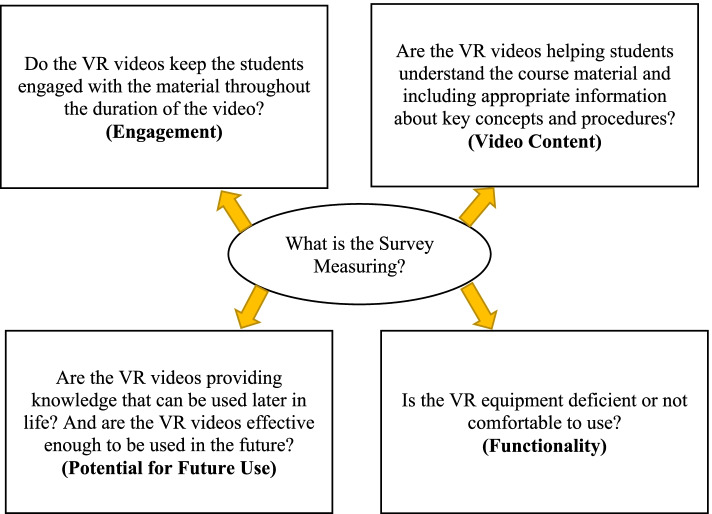
Details each aspect of the videos the survey questions are measuring

The survey distributed to the students after they experienced the VR lab videos included eleven 5-point Likert scale questions and seven open response questions. These survey questions were adapted from questionnaires used by Goehle (2018) (questions 4, 10, 11, 16, and 17) [5], Sultan et al. (2019) (questions 1, 2, and 4) [24], and Singh et al. (2020) (questions 6, 8, 13) [9]. Each survey question measured an aspect of the videos (engagement, video content, potential for future use, or equipment functionality) detailed in Fig. 3. The following tables (Tables 1 and 2) show which aspect each question was assigned. The survey was administered by the Qualtrics system.

**Table 1:** 5-point Likert scale questions

Questions	What is being measured?
1.The use of VR helped me feel more engaged with the lesson	Engagement
2.The use of VR allowed me to learn at my own pace	Engagement
3.The use of VR technology eliminated or reduced auditory and visual distractions from the environment	Engagement
4.The use of VR technology helped me understand the material	Video Content
5.The VR videos increased my retention of the course material	Video Content
6.The videos provided enough information to understand the task	Video Content
7.The use of videos met my expectations about this lab	Video Content
8.The videos provided an acceptable alternative to in-person labs	Potential for future use
9.I would feel confident applying the skills/techniques from the videos in person	Potential for future use
10.I would like to use this kind of video in future labs	Potential for future use
11.I experienced some kind of discomfort (e.g. claustrophobia, nausea, dizziness) while using the VR technology	Functionality

**Table 2:** Open response questions

Questions	What is being measured?
12.Was the length of the videos appropriate for the material covered? Please explain your answer	Engagement
13.Did you watch any of the videos multiple times? If so, why?	Video Content
14.Did you experience any problems using/viewing the videos for the lab? If so, which ones?	Functionality
15.Did you use the headset while watching the VR videos? Please explain	Functionality
16.What aspects of the VR lessons were helpful and/or effective?	N/A
17.What aspects of the VR lessons were not helpful nor effective?	N/A
18.Suggestions or comments	N/A

### Data analysis

The data collected from the survey was exported and graphed using Excel and analyzed.

For the Likert questions, percentages of each response were calculated. These distributions were graphed. Similar comments for the free-response questions were sorted and percentages were calculated. All questions were grouped according to the video aspects they measured to draw conclusions.

## Results

The surveys provided information about student feedback on VR videos as an educational tool. It was intended to measure student engagement, video content, the potential for future use, and equipment functionality.

### Engagement

**Fig. 4:**
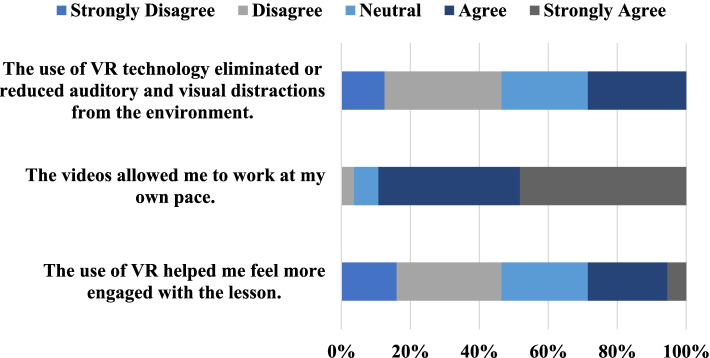
Student responses to the questions measuring engagement during the VR experience reported as a percentage

To measure engagement, 3 multiple choice questions were asked. These questions and the student response distributions are shown in Fig. 4.

From the student responses, it is observable that the VR videos were highly effective at allowing students to work at their own pace, with 48% of responses strongly agreeing and 41% agreeing with the statement. Only 4% of the students disagreed with the statement and no student responded strongly disagree. 7% were neutral to the statement. However, not many students agreed (23%) or strongly agreed (5%) with the statement that VR videos made them feel more engaged with the material, with 25% reporting a neutral response, 30% disagreeing, and 16% strongly disagreeing. Only 29% of students agreed that VR videos helped in reducing environmental distractions, with 25% reporting a neutral response, 34% reporting that they disagreed, and 13% reporting that they strongly disagreed with the statement. No students responded strongly agree.

There was one open response question measuring engagement that was included in the survey, namely, “Was the length of the videos appropriate for the material covered? Please explain your answer”.. 64% of the students thought the videos had an appropriate length. Some student opinions were “Yes, the videos were long enough to cover the necessary content, but not so long that it was hard to focus.” and “Yes, the videos covered all of the lab techniques”. 21% thought that only some videos had appropriate length. Finally, 14% thought that the length of the videos was not appropriate. Some students commented “I think they could be shorter to keep the audiences attention” and “A little too long to remember specific details when we couldn't refer back to it for quizzes.”

### Video content

**Fig. 5:**
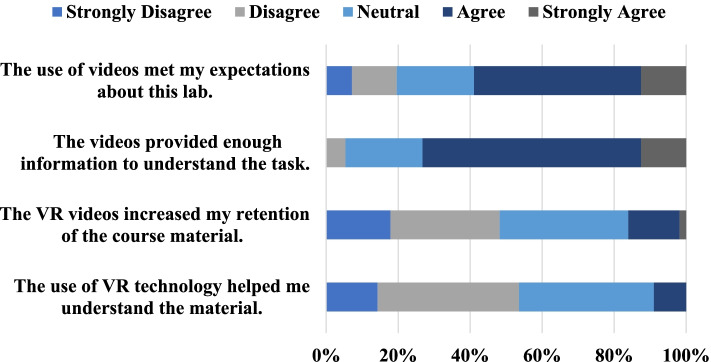
Student responses to the questions regarding the content of the VR videos, reported as a percentage

The quality of the information included in the VR videos was measured with 3 multiple-choice questions. The student response distributions of these questions are shown in Fig. 5.

Only 14% of students agreed with the VR videos helping them retain the course information, with 2% responding strongly agree, 36% responding neutral, 30% disagreeing, and 18% strongly disagreeing. Moreover, only 9% of students agreed that the VR was helpful in making them understand the material. 38% of students were neutral to this statement, 39% disagreed, and 14% strongly disagreed. No students strongly agreed. On the other hand, 61% of students agreed that the videos provided enough information to understand the course material with 13% strongly agreeing, 21% responding neutral, only 5% disagreeing, and no students responding strongly disagree. 46% of students agreed that the videos met their expectations for the remote lab. 13% of students responded strongly agree, 21% were neutral, 13% disagreed, and 7% strongly disagreed.

One open response question intended to measure the video content was included in the survey. This question is “Did you watch any of the videos multiple times? If so, why?”. It was important to know if the students re-watched the videos each time they did not understand the material. Also, watching the videos multiple times would increase the retention of the material, especially for visual learners. 68% of the students watched the video multiple times. 39% of those students re-watched the videos to prepare for the lab quizzes and reports. One of the students comment was “Yes. I watched some of the videos multiple times in order to review the material for the quizzes,”. 37% of those students re-watched the videos to understand the material better and increase retention. A student responded “Yes. To refresh myself on the content, and because I am a bit of a visual learner so the videos helped the information stick.” 11% of those students re-watched the videos because they missed important information during the first time they watched the VR videos. A student commented “Yes because sometimes I would accidentally miss some information that I would need.” Finally, 13% of those students re-watched the videos because it was difficult for them to hear, watch, or understand. A student said, “Yes, sometimes the audio was not clear.”

### Potential for future use

**Fig. 6:**
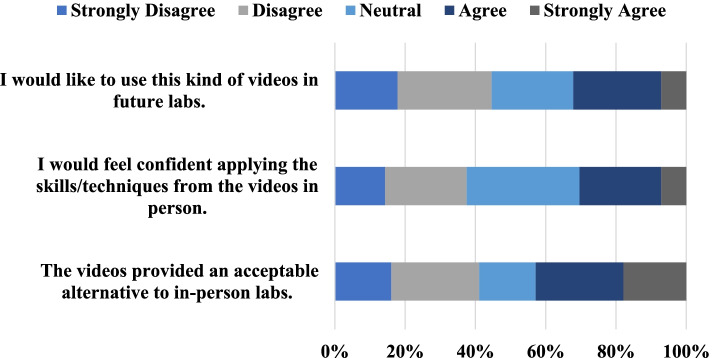
Student responses to the questions regarding the potential for future use of the VR experience, reported as a percentage

The survey contained 3 multiple choice questions that measured the potential for VR videos to be used in future courses. These questions and the response distributions are shown in Fig. 6.

Overall, 25% of students agreed that the videos were an acceptable alternative to in-person labs with 18% strongly agreeing, 16% responding neutral, 25% disagreeing, and 16% strongly disagreeing. 23% of students agreed and 7% strongly agreed on having the confidence of applying the learned skills to an in-person situation. 32% of students were neutral to this statement, 23% disagreed, and 14% strongly disagreed. Finally, 25% of students agreed and 7% strongly agreed on wanting VR videos in future labs. 23% of students were neutral, 27% disagreed, and 18% strongly disagreed.

Some students voiced support for the use of VR in remote labs including the following comments, “Overall, for an online lab, I thought this went pretty well. It obviously would have been nice to have this lab in person, but considering the circumstances, this was a good substitute,” “The videos were nice that I could move around in the video to focus on something in particular,” and “I think the videos were a fantastic idea to combat the virtual situation. I believe there can definitely be more improvements, but they are really close to being the best way to introduce lab material.” However, other students’ responses opposed the use of VR including the following comments: “The VR headsets were a good idea, but personally I found them to not be very useful,” “Honestly this was a good idea, but it just didn't work very well in practice. Better headsets and more work to make better videos would probably change a lot…” and “I would suggest that if a situation arises where labs have to be virtual in the future, VR should not be utilized.”

### Functionality

**Fig. 7:**
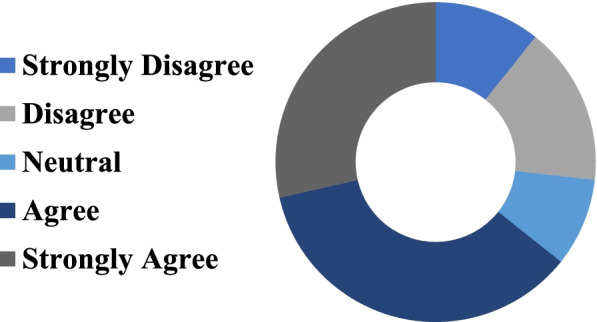
Student responses to the survey question “I experienced some kind of discomfort (e.g. claustrophobia, nausea, dizziness) while using the VR technology.”

One multiple choice question within the survey measured the functionality of the VR videos and equipment. This question and the responses are represented in Fig. 7.

36% of students agreed and 29% strongly agreed on having some kind of discomfort caused by the VR experience. 9% of students were neutral, 16% disagreed, and 11% strongly disagreed.

Two open response questions had the objective to measure technical features of the VR included in the survey. These questions are “Did you experience any problems using/viewing the videos for the lab? If so, which ones?” and “Did you use the headset while watching the VR videos? Please explain.”. It is important to make sure that the students experienced minimal or no technical issues to make the VR experience an effective educational tool.

From the first question, 62% of students did not experience problems viewing the VR videos and 38% of the students did experience problems. Out of that 38% of students, responses included 4 comments that said it was hard to hear or view parts of the videos. A student said, “The quality of the lens were too low and as a result the video was too blurry to actually see any fine detail.” 10 comments reported that the figures/text/images included in the videos were distorted, cut off, or hard to understand. A student remarked, “Yes, the VR device with the video would sometimes malfunction and it would distort some of the video images as well.” 4 comments mentioned experiencing headaches or dizziness while watching the videos. “Yes the VR made me feel sick” a student answered. 3 comments mentioned issues with the quality of the headsets. For example, a student said “Yes. I experienced double vision while using the headsets. I also experienced discomfort as a result of the nose piece from the VR headset.” 1 student commented on having difficulty using the desktop VR feature through YouTube. Finally, 1 student mentioned it was difficult to take notes while watching the VR videos.

From the second question, 54% of students did not use the headset while watching the VR videos. Out of that 54%, responses included 14 comments that the headset made them feel sick (headaches, dizziness). A student said, “No, I was unable to watch the videos using the VR set because I got really dizzy.” 3 comments mentioned that the quality of the headset made it uncomfortable to use them. A student remarked, “No, It was not very comfortable and I do not like the idea of a screen so close to my eyes.” Finally, 4 comments said that their phone had trouble fitting in the headset. A student mentioned “no, my phone was too big to fit into it properly”.

### Lab quiz scores

**Fig. 8:**
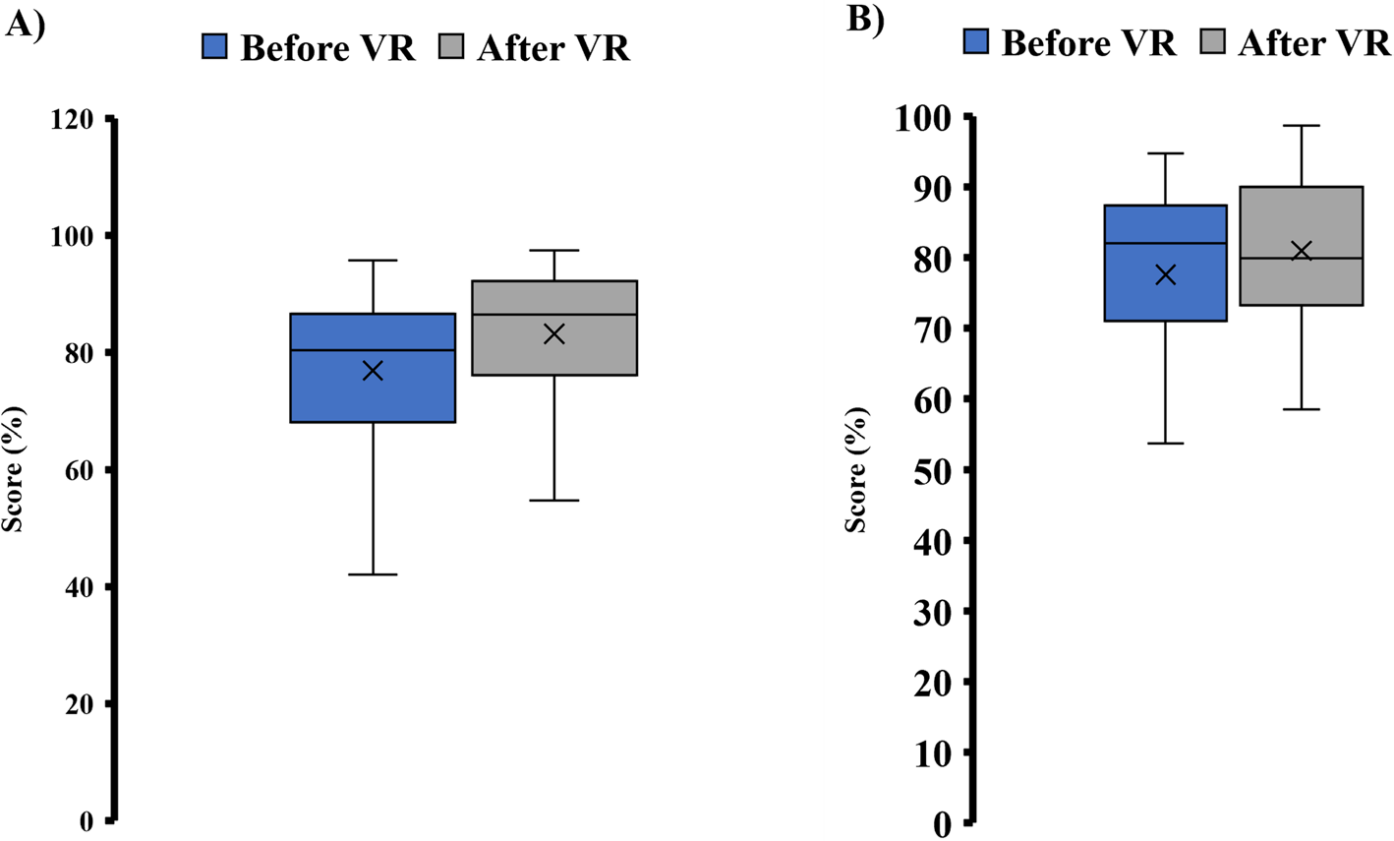
Box plot distributions of (**A**) pre-lab quiz scores in percentage and (**B**) post-lab quiz scores in percentage. * indicates statistical significance (*p* < 0.05)

The students’ pre-lab quiz scores for labs before the VR videos had an average of 76.90% with a standard deviation of 12.95%. The pre-lab quizzes for labs utilizing the VR videos provided an average score of 83.18% with a standard deviation of 11.72%. Figure 8a displays the pre-lab quiz score distributions. The students had an average of 77.59% for the post-lab videos for labs before the VR videos with a standard deviation of 13.65. The post-lab quizzes for labs utilizing the VR videos had an average score of 81.00% with a standard deviation of 11.07%. Figure 8b displays the post-lab quiz score distributions.

Paired t-tests (with α = 0.05, *n* = 56) were used to compare students’ pre-lab quiz scores for labs before and after VR labs and scores for the post-lab quizzes for labs before and after VR labs. For the t-test comparing the pre-lab scores, a p-value of 0.000004 was calculated showing a statistically significant difference in pre-lab quiz scores between labs before VR and the labs utilizing VR. The t-test comparing the post-lab quiz scores calculated a p-value of 0.025546, showing a statistically significant difference between the students’ scores on post-lab quizzes for labs before VR and the labs utilizing VR.

## Discussion

The results of this study suggest that VR videos could be an effective educational tool in a remote lab course but could be improved in various aspects.

With respect to student engagement, the videos could be improved by remaining within 10 to 20 min in length. Students reported it was difficult to remain focused for the duration of some of the lab videos. A better method would be to divide the videos up into multiple shorter videos with each video focusing on a particular skill or segment of the lab. This would help keep students’ attention for the full video and provide an opportunity for students to reflect or review what they just learned before moving on in the lab material. One student commented, “I thought all the videos that were between 10–20 min were the perfect length. The ones over that time frame were very hard to focus and pick up all the small details.”

The videos proved effective in allowing students to work at their own pace. This is important for keeping students engaged with the material as they have the option of re-watching or pausing the video at key points in the experiment. This feature not only provides students sufficient time to absorb the information and re-watch the videos later to prepare for an evaluation, but it also allows them to take breaks when they feel like their attention span has ended, making visual learning more effective. The Google Cardboard headsets did not prove to be effective in reducing distractions from the environment. This could be due to the Google Cardboard headsets not containing any audio capabilities as only visual distractions were removed. Sounds from the environment were still present unless students used headphones with the videos. This deficiency could have contributed to the students' mixed responses when asked if the VR videos helped the students feel more engaged with the material as it could have taken away from the students' sense of presence in the virtual environment. Additionally, students’ previous experience with VR technology, or lack of it, could factor into the student’s engagement with the material as students more familiar with VR may have been able to better concentrate on the videos.

Regarding the content of the VR videos, the videos were effective at providing the information needed for the lab. A student said, “I think explaining how to do the practical aspects of lab allowed for more effective understanding of what we were doing”. Overall, based on the answers to the open response question “Did you watch any of the videos multiple times? If so, why?” most of the students re-watched the VR videos because they thought it was a good way to study, explained the information well and in a visual way, and helped them retain or understand the information needed for the course. Participants commented, “The videos were done well and the TAs were good about giving information”, “The visual images added into the video and the captions explaining the procedure being demonstrated were helpful”, and “The visuals were useful to convey the procedures. I am a visual learner so it was easier to comprehend the material better by watching someone rather than reading a protocol sheet”.

The students’ opinions about the VR lab videos serving as an effective tool to use in future courses point out that most of the students prefer in-person labs. Practical experience develops skills that can be used in future courses, research, and industry, however, under circumstances when lab spaces are unavailable, the VR videos proved an adequate substitute. The results provide support that VR videos could be used in future courses with some improvements.

There was a significant difference between the pre-lab quiz scores for the labs before the VR videos and the labs utilizing the VR videos with the pre-lab quiz scores for labs utilizing the VR videos having a greater average score. Also, there was a significant difference between the post-lab quiz scores for the labs that did not utilize the VR videos and the labs with the VR videos. The increase in the lab quiz scores could be attributed to the VR videos providing the students with more interaction with the materials that contribute to the students’ focus on the content.

The quality of the videos can be improved to eliminate any difficulties in hearing, viewing, or understanding the material. Based on responses to the open response question “Did you experience any problems using/viewing the videos for the lab? If so, which ones?” better filming and editing of the videos is needed. A student mentioned, “It was often hard to see the TAs perform the actual experiments. It was also hard to hear when the camera was in the fume hood”. A quality microphone attached to the instructor would improve audio issues as the instructor would be heard more clearly over any background noise. This is especially important for videos recorded in a laboratory setting as there is often equipment being used that can produce a lot of background noise. Also, the images, figures, and texts need to be placed and scaled carefully within the VR environment to ensure they can be viewed properly in the VR videos. A student said, “Sometimes the images/text previewed on the screen were cut off and I could not read everything; also, images in VR always seemed compressed and hard to read”. Additionally, the camera should remain in one position, preferably on a tripod or otherwise not handheld, to ensure stability and prevent motion sickness effects. A 2D option needs to be available for students that feel sick when experiencing the VR as some students can be affected more than others.

Finally, help on VR installation and handling needs to be available for students who experience any problems. Based on responses to the open response question “Did you use the headset while watching the VR videos? Please explain” a better quality of headsets is needed for effective VR experiences and a 2D option needs to be available for students who experience some kind of discomfort when using the headset. Moreover, the headset needs to be adjustable for different phone sizes or an option without the need of using the student’s phone must be available. A student commented “I would suggest that in the future if VR is used that higher-quality headsets be used. The main reason that I disliked the headset was due to the discomfort I felt while using them.”

As it is observable, students would have preferred in-person labs. However, the situation did not give them the opportunity to experience that. As education returns to a non-virtual setting, VR alone should not replace in-person labs, but VR videos could be an effective educational tool if used in conjunction with in-person labs. Students would have the opportunity to experience the lab additional times or get information that they might have missed during the lab session while still getting the benefit of in-person hands-on experience.

The use of VR videos could also be a beneficial tool outside of laboratory settings. Studies involving primary children have shown the use of mobile VR technologies in synergy with traditional teaching methodologies improved the music learning experience in primary education, in terms of active listening, attention, and time [25]. Students in Singapore demonstrated significant improvement in molecular biology achievement after being exposed to 3D VR modules. The 3D modules helped students improve understanding, as well as stimulated interest and engagement [26].

Similar results were obtained by researchers at the University College Cork(UCC) in Ireland who developed virtual simulations for the teaching and learning of the spatial and structural complexity of viruses and next-generation molecular systems. Enhanced Active LEarning in Virology, cell culture, and molecular biotechnology (ELEVATE) developed a series of immersive VR simulations for the teaching of virus structure, recombinant plasmids, and green chemistry solutions for the bioeconomy. The team created pedagogically robust learning experiences with embedded assessments. A pilot survey (n = 22) completed by students taking a microbiology module at UCC prior to the codesign of the bespoke VR simulations showed that (a) 88% of respondents could see potential in the use of digital technologies, (b) 79% of respondents indicated that they learn well through visual modules and, (c) 15% of respondents declared competency in the use of VR technologies. Together, these data highlight the huge potential for VR integration into molecular biology curricula [27].

## Conclusions

This educational research was intended to determine if VR videos are an effective educational tool. Although the effectiveness of the VR videos is dependent on the quality of the video content, video editing, and equipment, it is possible to determine which aspects of the videos were more effective than others for future applications. The responses from the students that experienced the VR videos show that they were effective at providing the appropriate content and allowed students to work at their own pace. However, the quality of video filming, editing, and handling needs to improve for maximum educational effectiveness.

In the future, further studies should be conducted on the use of VR videos to supplement in-person learning. These studies would benefit from the use of higher quality equipment in both the camera to record the videos, and the headsets used by students. Dividing videos into multiple, shorter videos would help students stay focused on the material and may reduce students’ discomfort with the equipment. Additionally, scores for laboratory assignments, such as quizzes or lab reports, could be used to quantitatively analyze the effect of VR videos on student engagement with the material.

## Data Availability

Authors will make the study data available upon request while adhering to the IRB protocol and the human subjects consent forms.

## Abbreviations

VR: Virtual Reality
2D: Two-dimensional
3D: Three-dimensional
